# Evaluation of inhaler technique and achievement and maintenance of mastery of budesonide/formoterol Spiromax® compared with budesonide/formoterol Turbuhaler® in adult patients with asthma: the Easy Low Instruction Over Time (ELIOT) study

**DOI:** 10.1186/s12890-018-0665-x

**Published:** 2018-06-28

**Authors:** David B. Price, Vicky Thomas, P. N. Richard Dekhuijzen, Sinthia Bosnic-Anticevich, Nicolas Roche, Federico Lavorini, Priyanka Raju, Daryl Freeman, Carole Nicholls, Iain R. Small, Erika Sims, Guilherme Safioti, Janice Canvin, Henry Chrystyn

**Affiliations:** 1 Observational and Pragmatic Research Institute Pte Ltd, Singapore, Singapore; 2 University of Aberdeen, Aberdeen, UK; 3 Radboud University Nijmegen Medical Center, Nijmegen, The Netherlands; 4 Woolcock Institute, University of Sydney and Sydney Local Health District, Sydney, NSW Australia; 5 University Paris Descartes, Paris, France; 6 University of Florence, Florence, Italy; 7 Norwich Medical School, University of East Anglia, Norwich, UK; 8 Teva Pharmaceuticals Europe BV, Amsterdam, The Netherlands; 9 Inhalation Consultancy Ltd, Leeds, Yeadon UK

**Keywords:** Budesonide/formoterol, Dry-powder inhaler, Inhaler technique, Inhaler mastery, Intuitive, Pragmatic clinical trial, Asthma

## Abstract

**Background:**

Incorrect inhaler technique is a common cause of poor asthma control. This two-phase pragmatic study evaluated inhaler technique mastery and maintenance of mastery with DuoResp® (budesonide-formoterol [BF]) Spiromax® compared with Symbicort® (BF) Turbuhaler® in patients with asthma who were receiving inhaled corticosteroids/long-acting β2-agonists.

**Methods:**

In the initial cross-sectional phase, patients were randomized to a 6-step training protocol with empty Spiromax and Turbuhaler devices. Patients initially demonstrating ≥1 error with their current device, and then achieving mastery with both Spiromax and Turbuhaler (absence of healthcare professional [HCP]-observed errors), were eligible for the longitudinal phase. In the longitudinal phase, patients were randomized to BF Spiromax or BF Turbuhaler. Co-primary endpoints were the proportions of patients achieving device mastery after three training steps and maintaining device mastery (defined as the absence of HCP-observed errors after 12 weeks of use). Secondary endpoints included device preference, handling error frequency, asthma control, and safety. Exploratory endpoints included assessment of device mastery by an independent external expert reviewing video recordings of a subset of patients.

**Results:**

Four hundred ninety-three patients participated in the cross-sectional phase, and 395 patients in the longitudinal phase. In the cross-sectional phase, more patients achieved device mastery after three training steps with Spiromax (94%) versus Turbuhaler (87%) (odds ratio [OR] 3.77 [95% confidence interval (CI) 2.05–6.95], *p* < 0.001). Longitudinal phase data indicated that the odds of maintaining inhaler mastery at 12 weeks were not statistically significantly different (OR 1.26 [95% CI 0.80–1.98], *p* = 0.316). Asthma control improved in both groups with no significant difference between groups (OR 0.11 [95% CI -0.09–0.30]). An exploratory analysis indicated that the odds of maintaining independent expert-verified device mastery were significantly higher for patients using Spiromax versus Turbuhaler (OR 2.11 [95% CI 1.25–3.54]).

**Conclusions:**

In the cross-sectional phase, a significantly greater proportion of patients using Spiromax versus Turbuhaler achieved device mastery; in the longitudinal phase, the proportion of patients maintaining device mastery with Spiromax versus Turbuhaler was similar. An exploratory independent expert-verified analysis found Spiromax was associated with higher levels of device mastery after 12 weeks. Asthma control was improved by treatment with both BF Spiromax and BF Turbuhaler.

**Trial registration:**

EudraCT 2013-004630-14 (registration date 23 January 2014); NCT02570425.

**Electronic supplementary material:**

The online version of this article (10.1186/s12890-018-0665-x) contains supplementary material, which is available to authorized users.

## Background

Asthma is a worldwide chronic inflammatory disorder of the airways with major adverse effects on affected individuals’ quality of life [1]. Despite advances in therapy, suboptimal asthma control is commonplace and is associated with a substantial socioeconomic burden [2–6]. The causes of poor asthma control are multifactorial and include several patient-related factors. Smoking, poor treatment adherence, and device handling errors have all been shown to have a negative impact on asthma control [7–12].

Therapeutic efficacy of inhalation therapy is dependent on the drug(s) reaching the targeted areas of the lower lung [5]. There is a growing body of evidence suggesting that errors in inhaler technique can reduce drug delivery to the lungs and produce poor clinical outcomes [2, 10, 12–16]. Correct inhaler technique should therefore be considered an essential component in asthma management [5, 10, 14, 17] and has become an integral part of the management strategy set out by the Global Initiative for Asthma (GINA). However, despite the importance of patient training, many healthcare professionals (HCPs) themselves lack proficiency in correct inhaler use, are not confident in demonstrating proper inhaler technique and, consequently, patients are neither taught nor checked in follow-up visits [18–23]. As a result, poor inhaler technique continues to represent a barrier to achieving optimal asthma control among many patients with asthma, a situation that has not improved over the past 40 years [5, 14, 24–26].

Although the use of all inhalers involves techniques associated with the preparation of the device, inhalation and post-inhalation behaviors, some steps associated with the correct use of different inhalers are device-specific [2, 14, 27, 28]. The CRITIKAL study, which investigated the association between specific inhaler errors of different devices and asthma outcomes, enabled the identification of specific inhaler technique errors that were associated with lack of asthma control [16]. Other factors shown to be associated with poor asthma control when using the Turbuhaler® were not breathing out before an inhalation, not tilting the head or sealing the lips round the mouthpiece, not using a fast inhalation and no breath-holding after each inhalation [16].

Because inhaler technique errors have implications for disease control and HCPs do not always deliver inhaler technique education [29, 30], it is important that devices are as intuitive as possible. Additionally, many patients express preferences for inhaler devices that they are able to use easily and efficiently [5], but to date, only a few studies have investigated intuitiveness and the maintenance of device mastery with different inhalers by patients with asthma [31–33].

Combined use of an inhaled corticosteroid (ICS) and a long-acting β2–agonist (LABA) is considered to be recommended for patients with asthma at step 3 or higher of the GINA guidelines [1]. ICS/LABA fixed-dose combinations are available in a range of inhaler devices, including DuoResp® budesonide/formoterol (BF) Spiromax® and Symbicort® (BF) Turbuhaler. In Europe, these two products are approved for use in adult patients with asthma or chronic obstructive pulmonary disease where an inhaled ICS/LABA is indicated [34].

This Easy Low Instruction Over Time (ELIOT) study was a pragmatic trial designed to compare the intuitiveness of BF Spiromax and a real-life comparator, the BF Turbuhaler by assessing achievement and maintenance of correct inhaler technique (mastery), in adult patients with moderate-to-severe asthma.

## Methods

The ELIOT study was a pragmatic 12-week, 2-stage, multicenter, open-label, randomized, parallel-group study designed to compare the achievement and maintenance of inhaler technique in adults with asthma. It was conducted between May 2014 and March 2015 at 68 centers in the UK. The study was divided into two parts: a cross-sectional phase at Visit 1, followed by a 12-week longitudinal phase with Visit 2 at the end.

### Inclusion and exclusion criteria

Patients aged 18–75 years with a diagnosis of asthma in accordance with the GINA guidelines [1], as evidenced by the presence of an Asthma READ Code (UK diagnostic coding system), were eligible to participate in the study provided they were receiving step 3 or 4 therapy for asthma as defined by the British Thoracic Society (BTS) guidelines for persistent asthma [35]. Exclusion criteria included any previous use of BF Spiromax, use of the BF Turbuhaler device in the preceding 6 months, the presence of a significant chronic lower respiratory tract disease other than asthma, a significant asthma exacerbation, a prescription of oral corticosteroids or antibiotic treatment within the preceding 2 weeks.

### Study design and procedures

**Fig. 1:**
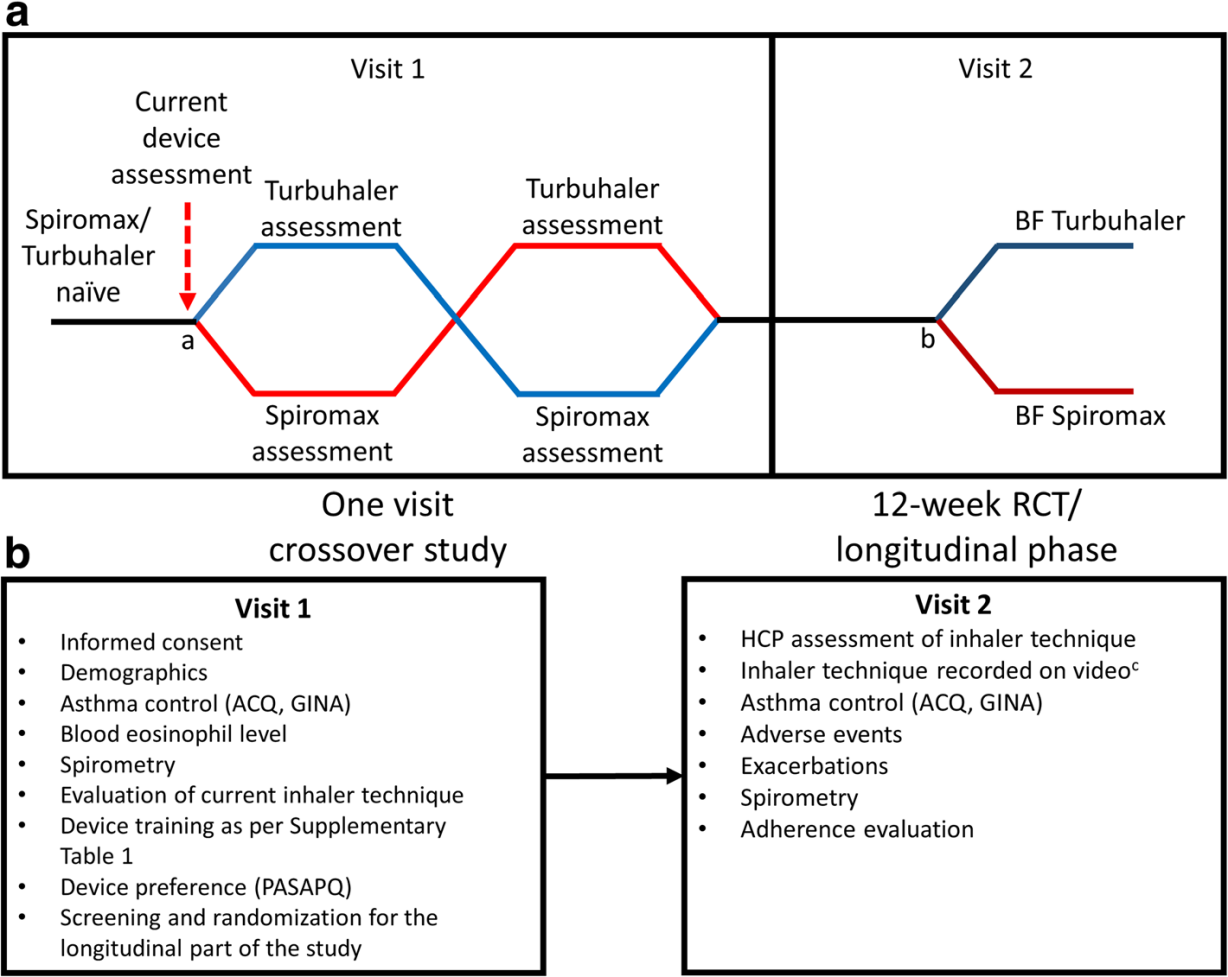
Study design (**a**) and procedures (**b**). ^a^Randomization into cross-over phase; ^b^Randomization into longitudinal phase; patients meeting the inclusion criteria: step 3 or 4 asthma therapy as defined by BTS guidelines for persistent asthma, partially or poorly controlled asthma as defined by GINA, and ≥ 1 error on current device were included; ^c^Video review was used only with patients who provided consent; patients not providing consent were able to continue in the study. ACQ: Asthma Control Questionnaire; BTS: British Thoracic Society; GINA: Global Initiative for Asthma; HCP: healthcare professional; PASAPQ: Patient Satisfaction and Preference Questionnaire; RCT: randomized control trial

As shown in Fig. 1, the study comprised an initial cross-sectional phase at Visit 1, followed by a 12-week longitudinal phase with Visit 2 at the end.

#### Cross-sectional phase

Prior to patient enrollment, HCPs conducting clinics involved in the study were trained to instruct patients on the correct use of the Spiromax and Turbuhaler devices. This included emphasizing the prescription dose (two inhalations, twice per day) and reminding patients to reload their device and take the second inhalation. This prior device training for HCPs was supported by a training manual and videos showing the proper use of each device as well as videos highlighting potential errors that patients could make when using them.

During Visit 1 (Fig. 1a) patients were evaluated on the handling of their current inhaler device and taught the proper use of empty Spiromax and Turbuhaler inhalers by the trained HCPs.

Assessments at this stage (Fig. 1b) included baseline demographic and clinical characteristics (including medical history, prior and concomitant medications), recording of a patient’s last peripheral blood eosinophil level (from medical records, if available), asthma review (completion of asthma review questionnaire and asthma control questionnaire [ACQ]), evaluation of current inhaler technique by HCP assessment and spirometry. Upon completion of these assessments, patients were randomized to receive device training with either empty Spiromax followed by empty Turbuhaler devices or vice versa. Randomization was accomplished using interactive response technology (IRT).

Details of how patients were trained in correct inhaler technique and how device mastery was determined and assessed are presented below.

##### Training on inhaler technique and device mastery

After randomization, each patient was trained to use the empty Spiromax and Turbuhaler devices using a six-step approach (details provided in Additional file 1: Table S1), as follows: (1) intuitive use (prior to any training); (2) after reading patient information leaflet (as supplied to patients in standard clinical practice); (3) following instructional video; (4) following tuition by HCP; (5) following first repeat of tuition by HCP; (6) following second repeat of tuition by HCP.

After each training step, an assessment of device use was carried out by HCPs using a predefined list of inhaler errors (Additional file 1: Table S2) collated from the best available evidence (developed through steering committee discussion and consensus). The predefined list was split into sections relating to dose preparation and the inhalation maneuver, with separate assessments being made for inhalations 1 and 2.

Device mastery was defined as the absence of HCP-observed errors. Once this had been attained or all six steps had been completed for the first inhaler device, patients were trained on the second device according to the randomly assigned order.

##### Recording of patients’ device preferences using the PASAPQ questionnaire

After device training was completed, patients’ preference for the Spiromax versus the Turbuhaler was assessed using the Patient Satisfaction and Preference Questionnaire (PASAPQ). The PASAPQ is a multi-item measure of respiratory inhalation device satisfaction and reference, designed and validated in patients with asthma and chronic obstructive pulmonary disease [36].

At the end of Visit 1, patients who met the required criteria for entry into the longitudinal phase of the study were then randomly assigned to receive treatment with a BF Spiromax or BF Turbuhaler at an equivalent dose to the patient’s current ICS and retrained on that device [35] (Fig. 1).

Patients were eligible to proceed to the longitudinal phase if they made at least one inhaler technique error on their existing device (i.e. the one they were using when they entered the study) and had demonstrated mastery of both the Spiromax and Turbuhaler devices during the cross-sectional phase.

#### Longitudinal phase

In this second phase of the study, patients were randomly assigned to BF Spiromax or BF Turbuhaler for 12 weeks. Randomization to treatment was undertaken separately from randomization to device training order during the crossover phase of the study, but using the same IRT system. Patients were asked to return to the study center to have their technique checked again at Visit 2, which took place 12 weeks after Visit 1. In addition, patients’ asthma control was followed-up by telephone at Weeks 4, 8 and 12 (no more than 3 days before Visit 2). During these calls, each patient self-reported the first 6 items of the ACQ, any adverse events (AEs) and use of concomitant medications to investigators who were blinded to the patient’s randomized treatment assignment.

The maintenance of device mastery was assessed at Visit 2, 12 weeks after Visit 1. For those patients who had previously consented to video recording, a video recording of the patient’s handling of the study device at Visit 2 was also made to enable additional review by an independent external device expert. Device mastery was defined as the absence of observed errors after 12 weeks of device use as assessed by trained HCPs, and by independent external experts viewing the video recordings.

Pulmonary function (forced expiratory volume in 1 s and forced vital capacity) was assessed by spirometry at Visit 2. Drug safety was monitored by AEs, recorded by the study investigator or a designee and coded according to the Medical Dictionary for Regulatory Activities (MedDRA version 17.0). Investigators were asked to rate the likelihood of an AE being related to the study medication as possibly, probably or definitely related. Treatment adherence was assessed by device dose counters which displayed the number of actuations remaining.

### Endpoints

#### Co-primary

The cross-sectional phase co-primary endpoint was the proportion of patients in the full-analysis set (FAS; see below) achieving device mastery after training step 3 (instructional video) with Spiromax compared with Turbuhaler.

The longitudinal phase co-primary endpoint was the proportion of patients in the full-analysis set maintaining device mastery based on HCP observations with BF Spiromax compared with BF Turbuhaler after 12 weeks of device use.

#### Secondary

For the cross-sectional phase, secondary endpoints were: the proportion of patients achieving device mastery by steps 1 and 2, the number of steps required to achieve device mastery, the number of HCP-observed errors, and the patient device preference (PASAPQ score).

For the longitudinal phase, secondary endpoints were: the proportion of patients maintaining mastery after 12 weeks of device use when split into dose preparation and inhalation maneuver steps, total number of observed errors (assessed by HCP and technology), number of technology-observed inhalation errors, number of handling errors, adherence, efficacy (as assessed by ACQ scores, time to treatment failure [change of treatment or treatment for exacerbation or infection], exacerbations, impact of maintaining device mastery on time to treatment failure, use of concomitant medications), and AEs.

#### Exploratory (longitudinal phase only)

The first exploratory analysis was maintenance of device mastery at 12 weeks as defined by the absence of external expert- (video-)observed errors. Additional exploratory analyses included the number and type of HCP- and external expert-observed errors; aspects of asthma control (including symptoms and exacerbations) and current inhaler errors; aspects of asthma control and current inhaler errors.

### Statistical analysis

The FAS comprised patients who completed the crossover stage of the study. Patient demographics and baseline characteristics were summarized using descriptive statistics. Outcomes for device mastery achievement and maintenance were compared using parametric or non-parametric tests, as appropriate. For variables measured on an interval or ratio scale, a t-test or Mann Whitney U-test (depending on the distribution of the variable) was used; for categorical variables a Pearson’s Chi-square (or Fisher’s exact test if sample sizes were too small) was used. The proportion of patients achieving device mastery was analyzed using a conditional logistic regression model to calculate an odds ratio (OR), 95% confidence interval (CI), and *p*-value to quantify any difference between devices. The proportion of patients achieving maintenance of device mastery was analyzed using logistic regression. Superiority was demonstrated if the logistic regression model showed that the proportion of patients maintaining device mastery was significantly greater (at the 5% level) with one device compared with the other. The number of steps taken to achieve mastery, counts of errors, and PASAPQ scoring were all analyzed using paired t-tests or Wilcoxon signed ranks tests, depending on the distributions of the count/score data. The number and type of HCP- and external expert- (video-)observed errors were tabulated and Cohen’s Kappa Coefficient was used to quantify agreement of error counts between the assessors (HCP and external expert). HCP-observed errors were analyzed using a negative binomial regression model. The frequencies of errors after 12 weeks were tabulated by device. To assess the maintenance of device mastery according to external expert review of video recordings, the proportion of patients maintaining mastery of device across treatment groups was compared using a Chi-square test.

#### Sample size

For the assessment of device mastery, a sample size of 477 patients was needed to achieve 90% power to detect a difference in proportions of 0.08 when the proportion of discordant pairs is expected to be 0.282 and the method of analysis is a McNemar’s test of equality of paired proportions with a 0.05 two-sided significance level [37, 38]. For the longitudinal phase, a two-group Chi-squared test would have 90% power to detect the difference between maintenance of device mastery of 61.2% for BF Turbuhaler [39] and maintenance of device mastery of 78.9% for BF Spiromax (OR 0.422) when the sample size in each group is 139.

#### Study populations

The FAS was the primary analysis set for all study assessments. For the cross-sectional phase, this included all randomized patients participating in the cross-sectional phase (cross-sectional phase intent-to-treat [ITT] population) who completed assessments on both study devices. The longitudinal phase FAS included all patients in the longitudinal phase ITT population who returned for assessment of maintenance of inhaler technique at Visit 2 (week 12), and who had at least 10 weeks of inhaler use to which they were randomly assigned. The safety population was specific to the longitudinal phase and included all patients randomly assigned to treatment and who received at least one dose of the study drug.

## Results

### Patients

**Fig. 2:**
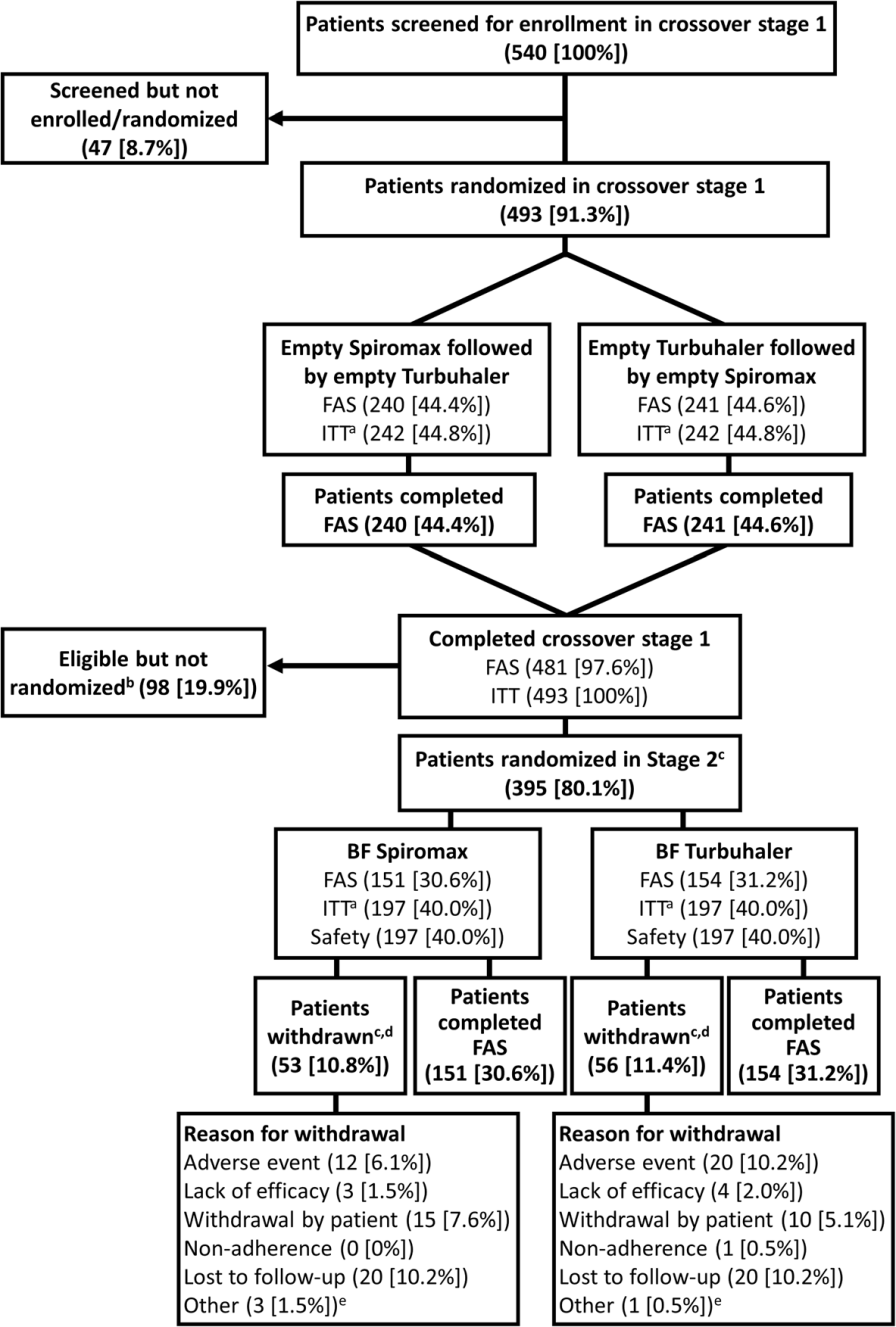
Patient disposition in the cross-sectional phase and the longitudinal phase. ^a^Eleven patients did not have device assessment because training devices were not available at the site. Withdrawals during the cross-sectional phase were not captured because of the study design. ^b^One patient was eligible for the longitudinal phase and was included in the ITT analysis; however, no device was available at the visit and the patient withdrew consent before treatment was allocated. ^c^Only patients who achieved device mastery on both devices could proceed to stage 2. ^d^Data were captured retrospectively. ^e^Other reasons for study withdrawal during the longitudinal phase included the following: Visit 2 was not scheduled and patient inclusion/exclusion criteria were not met. Note: Numbers in parentheses are numbers of patients. Percentages are based on the number of patients randomly assigned device order in the cross-sectional phase (*n* = 493). BF: budesonide formoterol; FAS: full analysis set; ITT: intent-to-treat

A total of 540 patients with asthma receiving step 3 or 4 treatment as defined by BTS guidelines were screened, 493 were enrolled and randomized, and 481 were included in the cross-sectional phase FAS (Spiromax followed by Turbuhaler, *n* = 240; Turbuhaler followed by Spiromax, *n* = 241) (Fig. 2). Eleven patients did not have device assessment because empty Spiromax and Turbuhaler devices were not available at the site, and one additional patient did not complete device assessment for reasons not recorded.

Prior to enrollment in this study, screened patients were using breath-actuated inhalers (Easi-breathe [*n* = 2], Autohaler [*n* = 3]), regular metered dose inhalers (*n* = 436), and dry powder inhalers (DPIs) (Diskus (*n* = 56), Novolizer (*n* = 1); prior inhaler use data were unavailable for 42 patients.

Of the 481 patients who completed the cross-sectional phase, 395 were randomized to the longitudinal phase and 305 were included in the longitudinal phase FAS (BF Spiromax, *n* = 151; BF Turbuhaler, *n* = 154) (Fig. 2). Of the latter group, 243 (80%) gave their consent to have their inhaler technique video-recorded; these patients were included in the FAS for the exploratory analysis, which entailed video review of their inhaler technique by an independent expert.

Patient demographics and baseline characteristics are summarized in Table 1. The most common comorbidity among participants in the cross-sectional phase was rhinitis (25% among patients randomized to use Spiromax followed by Turbuhaler; 24% in those randomized to Turbuhaler followed by Spiromax).

**Table 1:** Summary of baseline characteristics and demographics

	Cross-sectional phase			Longitudinal phase		
	Empty Spiromax followed by empty Turbuhaler (*N* = 243^a^)	Empty Turbuhaler followed by empty Spiromax (*N* = 242^a^)	*P*-value	BF Spiromax (*N* = 197^a^)	BF Turbuhaler (*N* = 197^a^)	*P*-value
Age, years, mean (SD)	54.4 (13.8)	53.1 (14.2)	0.309^b^	53.3 (14.3)	53.1 (14.1)	0.758^b^
Height, cm, mean (SD)	167.5 (9.1)	169.3 (9.4)	0.022^b^	168.1 (8.9)	168.8 (9.5)	0.348^b^
Sex, male, n (%)	91 (37.4)	108 (44.6)	0.108^c^	81 (41.1)	82 (41.6)	0.919^c^
FEV_1_, mean (SD)	2.5 (0.8)	2.6 (0.8)	0.238^b^	2.5 (0.8)	2.6 (0.9)	0.305^b^
ACQ-7, mean (SD)	1.4 (0.9)	1.6 (0.9)	0.043^b^	1.6 (1.0)	1.6 (0.9)	0.257^b^
Eosinophil levels, mean cells/nL (SD)	0.3 (0.3)	0.3 (0.3)	0.279^b^	0.3 (0.2)	0.3 (0.2)	0.595^b^
Medications, n (%)						
Prior medication	199 (81.9)	195 (80.6)	0.711^c^	179 (90.9)	184 (93.4)	0.350^c^
Concomitant medication	240 (98.8)	240 (99.2)	0.656^c^	194 (98.5)	196 (99.5)	0.315^c^

*ACQ-7* 7-item asthma control questionnaire, *BF* budesonide formoterol, *FEV*_*1*_ forced expiratory volume in 1 second, *SD* standard deviation

^a^Intent-to-treat population

^b^Mann-Whitney U Test

^c^Chi-Squared Test

### Device technique evaluations

#### Cross-sectional phase

The proportion of patients achieving device mastery (absence of errors) after step 3 (instructional video tuition) was significantly higher with Spiromax (94.4%) compared with Turbuhaler (86.9%; *p* < 0.001) (Table 2). Similarly, the proportion of patients achieving device mastery using the Spiromax versus the Turbuhaler in step 1 (intuitive use; 33.3% vs. 11.4%, respectively) and step 2 (after reading the patient information leaflet; 80.2% vs. 64.0%, respectively) was significantly higher with Spiromax compared with Turbuhaler. Furthermore, the number of steps taken to achieve device mastery and the number of HCP-observed errors were significantly lower with Spiromax compared with Turbuhaler (Table 2). Median PASAPQ scores (part 2 of the questionnaire) were statistically significantly higher for Spiromax versus Turbuhaler (89.8 vs. 85.7; *p* < 0.001). Higher levels of dose preparation mastery (97.3% vs. 92.1%; *p* < 0.001]) and inhalation maneuver mastery (96.3% vs. 92.5%, respectively; *p* = 0.007) at the end of step 3 were observed with Spiromax when compared with Turbuhaler (Table 2).

**Table 2:** Inhaler technique, device mastery and device preference in the cross-sectional phase

Device mastery	Empty Spiromax (*N* = 481)	Empty Turbuhaler (N = 481)	Odds ratio (95% CI)^a^	*P*-value
Step 1 (Intuitive use), n^b^ (%) [No. of patients participating]	160 (33.3) [481]	55 (11.4) [481]	4.89 (3.23–7.40)	< 0.001
Step 2 (Patient information leaflet), n^b^ (%) [No. of patients participating]	386 (80.2) [321]	308 (64.0) [426]	2.95 (2.06–4.22)	< 0.001
Step 3 (Instructional video), n^b^ (%) [No. of patients participating]	454 (94.4) [95]	418 (86.9) [173]	3.77 (2.05–6.95)	< 0.001
Achievement of dose preparation device mastery (primary endpoint; all patients), n (%)	468 (97.3)	443 (92.1)	4.12 (1.91–8.93)	< 0.001
Achievement of inhalation maneuver device mastery (all patients), n (%)	463 (96.3)	445 (92.5)	2.50 (1.28–4.88)	0.007
Number of steps taken to achieve device mastery, median (range)	1 (1–6)	2 (1–5)	N/A	< 0.001
Number of HCP-observed errors, median (range)	1.91 (0–6)	2.36 (0–6)	N/A	< 0.001
Patient device preference: PASAPQ score [scale of 1–100], median (range)	89.8^c^ (18.4–100)	85.7^c^ (14.3–100)	N/A	< 0.001

^a^Odds ratios are derived from conditional logistic regression of data from paired patients

^b^Numbers of patients achieving mastery at each stage are cumulative (i.e. patients achieving mastery at previous step(s) are included). Patients demonstrating mastery did not proceed to the next step; they were either proceeded to the second inhaler or finished the study (if they were using the second inhaler)

^c^*n* = 477 for each. *CI* confidence interval, *HCP*, healthcare professional, *N/A* not applicable, *PASAPQ* Patient Satisfaction and Preference Questionnaire

#### Longitudinal phase

Table 3 details the assessments of device mastery made during Visit 2 at the end of the longitudinal phase. The proportion of patients maintaining HCP-assessed device mastery over 12 weeks of device use was 58.9% with BF Spiromax and 53.2% with BF Turbuhaler; these values were not significantly different (*p* = 0.316). When the errors that impeded achievement of mastery were split into those relating to dose preparation and the inhalation maneuver, significantly fewer patients made dose preparation errors with Spiromax compared with Turbuhaler (*p* = 0.007) (Table 3).

**Table 3:** Maintenance of device mastery and inhalation errors after 12 weeks in the longitudinal phase

Outcome	BF Spiromax (*N* = 151)	BF Turbuhaler (*N* = 154)	Odds ratio (95% CI)	*P*-value^a^
HCP-observed errors				
Patients without errors (inhalations 1 & 2) (co-primary endpoint), n (%)	89 (58.9)	82 (53.2)	1.26 (0.80–1.98)^c^	0.316
Patients without errors related to dose preparation (inhalation 1), n (%)	128 (84.8)	111 (72.1)	2.16 (1.22–3.80)^c^	0.007
Patients without errors related to the inhalation maneuver (inhalation 1), n (%)	117 (77.5)	116 (75.3)	1.13 (0.66–1.92)^c^	0.657
Number of HCP-observed errors per patient, mean (SD)	0.50 (0.67)	0.81 (1.10)	0.61 (0.44–0.84)^d^	0.003
HCP-observed errors reassessed by video expert review (exploratory analysis)				
n^b^	119	124	–	–
Total number of errors observed by video expert	56	124	–	–
Patients without errors (inhalations 1 & 2), n (%)	79 (66.4)	60 (48.4)	2.11 (1.25–3.54)^c^	0.005
Number of video expert assessed errors per patient, mean (SD)	0.47 (0.78)	1.00 (1.26)	0.47 (0.34–0.64)^d^	< 0.001

*BF* budesonide formoterol, *CI* confidence interval, *HCP* healthcare professional, *SD* standard deviation

^a^Derived from Chi-squared test

^b^Note: Six patients for whom errors were reassessed by independent video review were not included in the full analysis set; data for these patients are not shown in the table

^c^Estimated from logistic regression models

^d^Estimated from negative binomial regression model

In an exploratory investigation, independent external experts assessed device mastery using video review of HCP-observed errors for 243 of the 305 (79.7%) patients participating in the longitudinal phase. Maintenance of mastery according to this analysis was significantly higher with Spiromax than with Turbuhaler (66.4% vs. 48.4%, respectively; *p* = 0.005), with patients observed to make less than half as many errors on average with Spiromax compared with Turbuhaler (0.47 vs. 1.00 errors per patient; *p* < 0.001]) (Table 3). The total numbers of HCP- and video-assessed errors per patient were also significantly lower with BF Spiromax compared with BF Turbuhaler (Table 3).

The HCP assessments during Visit 2 of each error (described in Additional file 1: Table S1) are shown in Table 4. The most common errors made with both inhalers were not inhaling fast enough from the start (14–17% of patients) and incorrectly shaking the inhaler before or after dose preparation (12–13% of patients). Nineteen patients (12.3%) using Turbuhaler failed to twist and/or untwist the base correctly, an action that is not required for Spiromax dose preparation. Errors specific to preparation of the second dose were made by 9–10% of patients using both inhalers.

**Table 4:** Frequency of HCP-observed errors after 12 weeks of device use in the longitudinal phase

	BF Spiromax (*N* = 151)	BF Turbuhaler (*N* = 154)
Number of patients with at least one error, n (%)	62 (41.1%)	72 (46.8%)
Total errors, n	75^a^	125^a^
Does not hold the inhaler with the semi-transparent mouthpiece cover at the bottom	0	N/A
Spiromax: Does not open cap Turbuhaler: Does not remove cap	0	0
Dose preparation: A click is not heard when the cap is opened	0	N/A
Dose preparation: not twisting the base as far as possible, until it clicks and not turning it back to the original position	N/A	19 (12.3%)
Spiromax: Inhaler is not held upright when a dose is prepared (±90° is acceptable) Turbuhaler: Inhaler is not held upright (mouthpiece skywards ±45°) when a dose is prepared throughout dose preparation	4 (2.6%)	8 (5.2%)
Turbuhaler: Device not held upright (upright means mouthpiece pointed skywards ±45°) after the base is twisted until inhalation	N/A	8 (5.2%)
Vigorous shaking before or after dose preparation	19 (12.6%)	18 (11.7%)
Exhales into the inhaler before taking dose	8 (5.3%)	13 (8.4%)
Fails to put in mouth and seal lips around mouthpiece	1 (0.7%)	1 (0.6%)
Spiromax: Finger (or face) placed over the air inlet during an inhalation (at front above the mouthpiece) Turbuhaler: Fingers or mouth placed around air inlets (positioned around the base and above the mouthpiece)	2 (1.3%)	10 (6.5%)
Inhalation is not as fast as possible (from the start)	25 (16.6%)	22 (14.3%)
Errors involving a second dose error, n		
Spiromax: Does not close cap after the inhalation and load a new dose; a click is not heard when the cap is opened Turbuhaler: Does not load a new dose as described; dose preparation: not twisting the base as far as possible, until it clicks and not turning it back to the original position	14 (9.3%)	15 (9.7%)
Spiromax: Does not close the inhaler after taking the second dose Turbuhaler: Does not place the cap back on the inhaler after taking the second dose	1 (0.7%)	7 (4.5%)
Does not know how to read out the dose counter after asking the patient to check the number of doses left	0	4 (2.6%)

*BF* budesonide formoterol, *HCP* healthcare professional, *N/A* not applicable

^a^Cohen’s Kappa Coefficient (measure the inter-rater (HCP and external) agreement of error) = − 0.606

Treatment adherence, assessed using device dose counters, showed no statistically significant difference in adherence when using the BF Spiromax inhaler versus BF Turbuhaler. For both regimens, adherence was ≤50% in 40% of patients. Adherence to treatment was in the range 51–70% for 13 and 10% of patients with Spiromax and Turbuhaler, respectively, in the range 71–99% for 46% of patients with both devices and reached 100% for just 1 and 4% of patients with these respective devices. Taking adherence into account, the average ICS daily dose in both groups was lower than the 800 μg/day inclusion criterion: 765 μg/day among BF Spiromax and 740 μg/day among BF Turbuhaler users who returned all of their devices (*n* = 227). All but nine patients returned at least one partially or completely full device.

### Efficacy and safety

Asthma control improved after 12 weeks of treatment in both treatment groups: mean changes in the 7-item ACQ from baseline to week 12 showed no statistically significant differences between BF Spiromax (− 0.20) and BF Turbuhaler (− 0.31) (OR [95% CI]: 0.11 [− 0.09–0.30]; *p* = 0.278). Blood eosinophil levels were generally not correlated with baseline ACQ, asthma control, or inhaler errors (Spearman’s correlation < 0.3).

Over 12 weeks of treatment, 57% of patients using BF Spiromax and 60% of patients using BF Turbuhaler experienced at least one AE (Table 5), most of which were mild or moderate in severity and deemed unrelated to treatment. The incidences of events considered by investigators to be possibly, probably or definitely related to study medication were 6.1% in the BF Spiromax group and 9.1% in the BF Turbuhaler group. Serious AEs were reported in 12 patients (4 on BF Spiromax and 8 on BF Turbuhaler) (Table 5; Additional file 1: Table S3), but these were generally deemed to be unrelated to study treatment. One serious AE (worsening of asthma, in a patient who received BF Spiromax) was considered by the investigator to be related to the study treatment. Withdrawals due to AEs were similar in the two study groups; the AEs most frequently causing withdrawal were cough, dyspnea, oral candidiasis, and worsening of asthma or asthma exacerbation.

**Table 5:** AEs occurring in ≥ 2% of patients

	BF Spiromax (*N* = 197)	BF Turbuhaler (*N* = 197)
Patients with at least 1 AE, n (%)	113 (57.4)	119 (60.4)
Infections and infestations		
Lower respiratory tract infection	17 (8.6)	31 (15.7)
Urinary tract infection	5 (2.5)	2 (1.0)
Nervous system disorders		
Headache	5 (2.5)	2 (1.0)
Respiratory, thoracic, and mediastinal disorders		
Asthma (worsening of asthma or asthma attack)	8 (4.1)	9 (4.6)
Cough	11 (5.6)	12 (6.1)
Dyspnea	5 (2.5)	5 (2.5)
Wheezing	4 (2.0)	3 (1.5)
Number of patients with at least 1 serious AE, n (%)	4 (2.0)	8 (4.1)

*AE* adverse event, *BF* budesonide formoterol

## Discussion

In this two-part pragmatic study, Spiromax was associated with higher levels of device mastery compared with Turbuhaler after training in the initial crossover phase. During this phase, patients were significantly less likely to make an HCP-observed error with the empty Spiromax device compared with the empty Turbuhaler device in intuitive use, after reading the patient information leaflet, and after viewing an instructional video. In addition, patients using the empty Spiromax achieved device mastery in fewer steps and made fewer errors overall.

In the longitudinal phase of the study, the proportion of patients maintaining device mastery (absence of HCP-observed errors upon assessment after 12 weeks of device use) was numerically higher with BF Spiromax than with BF Turbuhaler, but not statistically significantly so. However, in the exploratory analysis wherein an independent external expert viewed video recordings of participants’ inhaler usage, higher levels of mastery than were reported by the HCPs were reported, suggesting that HCPs may have had a tendency to over-estimate the number of errors.

The findings of the cross-sectional phase support the hypothesis that patients may find it easier to attain mastery of Spiromax compared with Turbuhaler. Fewer errors were observed with both devices when patients received tuition by reading the patient information leaflet and watching the instructional video (step 3; verbal instruction and demonstration given) than after reading the leaflet alone (step 2; no verbal instructions given). Device mastery at step 2 was achieved in a significantly greater proportion of patients using Spiromax versus Turbuhaler, but rates of achievement of device mastery were markedly increased following viewing of the instructional video at step 3.

In a ‘real-world’ setting, many patients may not have access to video instructions, or they may be of variable quality. In contrast, all patients should receive the patient leaflet, so maximizing patient mastery of inhaler technique using this resource is an important objective. The outcomes of this study are in agreement with previously published findings that passive instructions alone (such as reading the patient information leaflet) are not as effective as verbal instructions or demonstration (such as watching an instructional video) in teaching patients correct inhaler technique, specifically in patients using inhalers for the first time [40–43]. Other instructional tools for the training of correct inhaler technique should be considered, including verbal instructions and multimedia educational materials (e.g. a link to a website-based instructional video could be included in the patient information leaflet). Demonstrations and practice sessions should also be considered in clinical practice to maximize the number of patients with correct inhaler technique [5, 31, 32, 41, 44–47]. Overall, in order to improve device mastery, HCPs should consider a range of factors including the inhaler type, the patient, the educator/teacher, and the method of teaching [48].

Possible reasons why the longitudinal phase mastery results (based on HCP-observed errors) did not demonstrate a significant difference between the two devices include the fact that many longitudinal phase participants had previous experience of using the Turbuhaler (albeit more than 6 months prior to the study), whereas none had used the Spiromax. Cross-sectional studies [16, 24, 25] have reported a much higher frequency of errors with Turbuhaler than that observed in this study. Another possible explanation is the frequent telephone follow-up of patients, a situation rather different from normal clinical practice, which could have had an impact on the proportion of patients maintaining correct technique. Maintenance of correct device technique may be influenced by patient motivation. Previous studies, in which patients received intensive inhaler technique education in a community pharmacy setting, and had their technique checked 1 month later back at the pharmacy, showed correct technique in about 45 to 60% of patients at this time [31, 45].

It could also be argued that HCP assessment is prone to human observational errors. The discrepancy between HCP-reported error rates and those obtained through independent expert assessment of video-recorded use that was observed in our exploratory analysis suggests that HCPs may be conservative with patient assessments or lack experience and require training in assessment themselves, potentially resulting in over-estimation of error rates. In real-life clinical settings, it could be beneficial for external experts to review each patient’s inhaler technique with HCPs. The implementation of such assessment in busy clinics would be a challenge, and if this is not feasible, HCPs should ensure that they maintain their own inhaler technique mastery and periodically review each other’s technique. In the future, smartphone apps (including video recording of inhalation technique at home or augmented reality facial mapping) could become an option, as well as new technologies incorporating electronic sensors to inhalers. This study therefore highlights the potential for technology-assisted methods to enhance teaching and assessment of inhaler technique. Our findings also suggest that HCPs should receive training on inhaler technique and its evaluation, to ensure that they monitor their patients in a standardized manner reflecting the approach of an inhalation device expert.

Another important aspect of this study was the analysis of specific aspects of device mastery such as dose preparation. Spiromax was associated with significantly higher levels of dose preparation mastery in the cross-sectional phase compared with Turbuhaler and significantly fewer HCP-observed errors after the longitudinal phase. Errors in dose preparation can significantly impair effective delivery of drug to the lungs, leading to a risk of inadequate treatment dosing (or even no drug dose) [5, 8, 14, 46]. Previous studies have reported that dose preparation errors are device-specific whereas errors in inhalation maneuvers are usually observed across different devices of a particular type (in this study, both devices were DPIs) [24, 25, 49]. Studies in which dose preparation errors have been specifically examined report error rates of between 24 and 49% with Turbuhaler in patients who use this device for their regular medication [16, 49] and in 16.7% of people who are using Turbuhaler for the first time and have read the patient leaflet [43]. The findings of the latter study are consistent with the outcomes from our research, in that both studies found that (1) Spiromax was associated with a lower rate of dose preparation errors compared with Turbuhaler, and (2) maximal device mastery is achieved after HCP education and demonstration [43]. Research shows that when patients learn correct inhaler technique and adhere to treatment for 7 days, they are more likely to maintain device mastery over time [50].

It has recently been shown that not using a fast inhalation when using dry powder inhalers is one of the principal errors related to a greater incidence of poor asthma control [16]. Dose emission from a DPI is affected by inhalation flow, but the extent of variability differs between devices, so that it may be beneficial to use an inhaler with low variability [51].

Participants were instructed to take two inhalations twice daily, and the preparation of the second dose formed an important part of the device mastery assessment. Many of the inhalation 2 errors were due to patients making two inhalations from the first dose that was prepared, instead of preparing a second dose. They made a second inhalation either without closing and opening the cap of the Spiromax inhaler or without rotating the base of the Turbuhaler. Patients were observed to make this type of error with both Spiromax and Turbuhaler with a similar frequency, suggesting that they may have misinterpreted the dosing instructions or had never properly been instructed. Further studies are therefore required to investigate if this is a problem that needs to be addressed. The role of HCPs in ensuring that patients are fully aware of all steps required, including those that apply when taking a second dose if applicable, and directing patients to suitable training material, must therefore be emphasized.

Patients are more likely to prefer a device that they consider easy to use and that they can use correctly, and patient satisfaction with an inhaler is correlated with clinical outcomes [5, 10, 17, 44, 48, 52]. Hence, selecting the correct inhaler device by matching the patient with the inhaler that they prefer and find more intuitive to use could potentially lead to improved adherence, better clinical outcomes and reduced costs for care [5, 13, 28, 48, 53].

Although the between-group difference was small, patient-reported PASAPQ scores for Spiromax and Turbuhaler were significantly in favor of the empty Spiromax (PASAPQ score 89.8 vs. 85.7 for Turbuhaler; *p* < 0.001), indicating higher device preference with Spiromax. Similar findings were reported in two recent studies [43, 54]. One of these was a clinical study comparing BF Spiromax with BF Turbuhaler in patients with asthma; PASAPQ scores showed that patients preferred the Spiromax device for its performance and that they were more willing to continue therapy with Spiromax beyond the 12-week study period [54]. In the other study, device preference questionnaire results from inhaler-naïve Finnish volunteers showed that the majority of participants found Spiromax easier to use than Easyhaler or Turbuhaler and, if they were prescribed an inhaler, they would prefer Spiromax [43]. In our study, device preference was only assessed in the cross-sectional phase. It would be relevant to know whether device preference or satisfaction was maintained after 12 weeks of using the active inhaler (i.e. by repeating the PASAPQ at the end of the study and comparing the outcomes with the cross-sectional phase).

One strength of the current study is the assessment of the maintenance of device mastery, which has been addressed in very few other pragmatic studies in asthma [31, 32, 45]. This begins to address a gap in the literature and provides a preliminary insight into the possible impact of inhaler technique and mastery on disease control. For a formal assessment, adjustment for confounding factors such as adherence will be required. Our study suggests that maintaining good inhaler technique could provide benefits to patients receiving high-dose ICS medication, as the daily dose of beclomethasone dipropionate-equivalent ICS in patients enrolled in this study was 800–2000 μg (fixed or free combinations with LABA). Dose modification and alternative choices for improving asthma control are generally limited in these patients. Another strength of this pragmatic study was the assessment of patients in real-life scenarios. In general, randomized clinical trials (RCTs) provide evidence from narrowly defined patient groups representing subsets of the real-life patient population; co-morbidities and lifestyle factors are not usually captured. Findings from pragmatic trials provide additional insight from a broader asthma population and can be combined with findings from conventional RCTs to provide a comprehensive picture of treatment efficacy and outcomes [55]. Among the limitations of this study are the open-label design, potential confounding associated with 4-weekly follow-up acting as a reminder to patients, and the subjective nature of some assessments such as HCP observations making it inherently prone to a degree of bias and personal judgment. The 4-weekly follow-up calls, in particular, may have had a positive influence on patients’ device mastery, as previous research has shown that regular follow-up of patients affects device mastery over time [31, 50]. A regimen requiring two doses to be taken twice daily also represents a dosing frequency greater than that typically found in the clinic, where patients more usually administer one dose, twice daily. In addition, the findings are not necessarily applicable to mild asthma which was outside of the scope of this study.

## Conclusions

In the cross-sectional phase of this study, Spiromax was associated with higher levels of device mastery compared with Turbuhaler in adult patients with moderate-to-severe asthma. No significant overall difference between the devices was observed by HCP assessment of inhaler technique in the longitudinal phase, though Spiromax users were observed to make fewer dose preparation errors than those using Turbuhaler. An exploratory investigation involving external expert assessment of inhalation technique by video reported higher levels of maintenance of device mastery during the longitudinal phase with Spiromax versus Turbuhaler. Improvements in asthma control, similar with both devices, were observed over 12 weeks of BF treatment, despite low adherence levels in both study groups. In accordance with previous studies, device preference was higher with Spiromax than with Turbuhaler. This study supports the use of active (e.g. verbal) training to minimize the likelihood of inhalation errors. Our findings also highlight the possible value of independent assessment using video to assist HCPs in evaluating patients’ device mastery.

## Additional file

Additional file 1: **Table S1.** Training on inhaler technique using a six-step process* during the cross-sectional phase, Supplementary material – study governance, training on inhaler technique using a six-step process during the cross-sectional phase, checklist for inhaler errors, serious adverse events by preferred term. **Table S2.** Checklist for inhaler errors. **Table 3.** Serious adverse events by preferred term. (DOCX 20 kb)

## Abbreviations

ACQ: Asthma control questionnaire
AE: Adverse event
BF: Budesonide/formoterol
BTS: British Thoracic Society
CI: Confidence interval
DPI: Dry powder inhaler
ELIOT: Easy Low Instruction Over Time
FAS: Full-analysis set
GINA: Global Initiative for Asthma
HCP: Healthcare professional
ICS: Inhaled corticosteroid
IRT: Interactive response technology
ITT: Intent-to-treat
LABA: Long-acting β2–agonist
OR: Odds ratio
PASAPQ: Patient Satisfaction and Preference Questionnaire
RCT: Randomized clinical trial
