# Supplementary material for: Evaluation of inhaler technique and achievement and maintenance of mastery of budesonide/formoterol Spiromax® compared with budesonide/formoterol Turbuhaler® in adult patients with asthma: the Easy Low Instruction Over Time (ELIOT) study
Source: BMC Pulm Med. 2018 Jun 28;18:107. doi: 10.1186/s12890-018-0665-x (PMC6022306; doi:10.1186/s12890-018-0665-x)
Supplement: Supplementary file 1 — Table S1. Training on inhaler technique using a six-step process* during the cross-sectional phase, Supplementary material – study governance, training on inhaler technique using a six-step process during the cross-sectional phase, checklist for inhaler errors, serious adverse events by preferred term. Table S2. Checklist for inhaler errors. Table 3. Serious adverse events by preferred term. (DOCX 20 kb) [file 12890_2018_665_MOESM1_ESM.docx]

# Supplementary material

**Study governance**

Before the study was initiated, the protocol was submitted to the Independent Ethics Committee (IEC) or Institutional Review Board (IRB) according to national or local regulations. Any substantial protocol amendments were also submitted.

A list of all IECs/IRBs consulted, with their assurance numbers or committee chairpersons, can be provided upon request.

This study was conducted in full accordance with the International Conference on Harmonisation (ICH) Good Clinical Practice (GCP) Consolidated Guideline (E6) and any applicable national and local laws and regulations (e.g. Code of Federal Regulations [CFR] Title 21, Parts 50, 54, 56, 312, and 314; European Union [EU] Directive 2001/20/EC on the approximation of the laws, regulations and administrative provisions of the Member States relating to the implementation of GCP in the conduct of clinical trials on medicinal products for human use). Information regarding any investigational study centers participating in this study that could not comply with these standards was documented.

Each investigator was responsible for performing the study in accordance with the protocol, ICH guidelines, and GCP, and for collecting, recording, and reporting the data accurately and properly. Agreement of each investigator to conduct and administer this study in accordance with the protocol was documented in separate study agreements with the sponsor and other forms as required by national authorities in the country where the study center is located.

The principal investigator at each study center was responsible for the conduct and administration of the study at that center and for contacts with study center management, with the IEC/IRB, and with local authorities.

**Supplementary Table 1 Training on inhaler technique using a six-step process* during the cross-sectional phase**

| Step | Instruction | Detail |
| --- | --- | --- |
| 1 | Intuitive use | Patient was given the device and asked to demonstrate how to use it. No additional instructions were given. |
| 2 | Patient information leaflet | Patient was given the patient information leaflet provided with the device and after reading of the leaflet the patient was asked to demonstrate how they would use the device. |
| 3 | Instructional video | Patient was asked to watch a video on how to use the device and then demonstrate how they would use the device. |
| 4 | Tuition by HCP | Using pre-scripted instructions, the patient was shown how to use the device by the HCP. The patient was then asked to demonstrate how they would use the device. |
| 5 | Tuition by HCP (first repeat) | Using pre-scripted instructions, the patient was shown how to use the device by the HCP with focus on errors observed at step 4. The patient was then asked to demonstrate how they would use the device. |
| 6 | Tuition by HCP (second repeat) | Using pre-scripted instructions, patients was shown how to use the device by the HCP with focus on errors observed at steps 4 and 5. The patient was then asked to demonstrate how they would use the device. |

*Those making an HCP-assessed error at each step progressed to the next step.

HCP: healthcare professional

**Supplementary Table 2 Checklist for inhaler errors**

| **Spiromax** |
| --- |
| INHALATION 1 |
| *Dose preparation* |
| Does not hold the inhaler with the semi-transparent mouthpiece cover at the bottom |
| Does not open cap |
| A click is not heard when the cap is opened |
| Vigorous shaking before or after dose preparation |
| Inhaler is not held upright after dose preparation (±90 degrees is OK) |
| *Inhalation maneuver* |
| Exhales into the inhaler before taking dose |
| Fails to put in mouth and seal lips around mouthpiece |
| Inhalation is not as fast as possible (from the start) |
| Puts finger (or face) over the air inlet during an inhalation (at front above the mouthpiece) |
| INHALATION 2 |
| Does not close cap after the inhalation and load a new dose; a click is not heard when the cap is opened |
| Does not close the inhaler after taking the second dose |
| **Turbuhaler** |
| INHALATION 1 |
| *Dose preparation* |
| Device not held upright (upright means mouthpiece pointed skywards ±45 degrees) after the base is twisted until inhalation |
| Does not remove cap |
| Not twisting the base as far as possible, until it clicks, and not turning it back to the original position |
| Inhaler is not held upright when a dose is prepared (upright means mouthpiece skywards ±45 degrees) throughout dose preparation |
| Vigorous shaking before or after dose preparation |
| *Inhalation maneuver* |
| Exhales into the inhaler before taking dose |
| Fails to put in mouth and seal lips around mouthpiece |
| Inhalation is not as fast as possible (from the start) |
| Puts fingers or mouth around air inlets (positioned around the base and above the mouthpiece) |
| INHALATION 2 |
| Does not load a new dose as described; dose preparation: not twisting the base as far as possible, until it clicks, and not turning it back to the original position |
| Does not place the cap back on the inhaler after taking the second dose |

**Supplementary Table 3 Serious adverse events by preferred term**

| **n (%)** | **BF Spiromax (N = 197)** | **BF Turbuhaler (N = 197)** |
| --- | --- | --- |
| **Cardiac disorders** | | |
| Angina pectoris | 1 (0.5) | 0 (0.0) |
| Atrial flutter | 0 (0.0) | 1 (0.5) |
| Atrioventricular block | 0 (0.0) | 1 (0.5) |
| **General disorders and administration site conditions** | | |
| Chest pain | 0 (0.0) | 2 (1.0) |
| **Infections and infestations** | | |
| Lower respiratory tract infection | 1 (0.5) | 1 (0.5) |
| Postoperative wound infection | 1 (0.5) | 0 (0.0) |
| **Injury, poisoning, and procedural complications** | | |
| Thermal burn | 0 (0.0) | 1 (0.5) |
| **Renal and urinary disorders** | | |
| Renal failure acute | 0 (0.0) | 1 (0.5) |
| **Reproductive system and breast disorders** | | |
| Ovarian mass | 0 (0.0) | 1 (0.5) |
| **Respiratory, thoracic, and mediastinal disorders** | | |
| Asthma (worsening of asthma or asthma attack) | 2 (1.0) | 1 (0.5) |
| Pneumothorax | 0 (0.0) | 1 (0.5) |
| **Vascular disorders** | | |
| Hypotension | 0 (0.0) | 1 (0.5) |

BF: budesonide formoterol
